# Frequency and Characteristics of Injuries and Rehabilitation Procedures in Rugby Players in Poland and France

**DOI:** 10.3390/ijerph18094835

**Published:** 2021-04-30

**Authors:** Anna Lipert, Paweł Rasmus, Michał Marczak, Remigiusz Kozłowski, Anna Jegier, Małgorzata Timler, Dariusz Timler

**Affiliations:** 1 Department of Sports Medicine, Medical University of Lodz, 92-213 Lodz, Poland; anna.jegier@umed.lodz.pl; 2 Department of Medical Psychology, Medical University of Lodz, 90-131 Lodz, Poland; pawel.rasmus@umed.lodz.pl; 3 Department of Management and Logistics in Healthcare, Medical University of Lodz, 90-131 Lodz, Poland; michal.marczak@umed.lodz.pl (M.M.); malgorzata.timler@stud.umed.lodz.pl (M.T.); 4 Center of Security Technologies in Logistics, Faculty of Management, University of Lodz, 90-237 Lodz, Poland; remigiusz.kozlowski@wz.uni.lodz.pl; 5 Department of Emergency Medicine and Disaster Medicine, Medical University of Lodz, 92-213 Lodz, Poland; dariusz.timler@umed.lodz.pl

**Keywords:** injuries, rugby, rehabilitation, injury incidence, training

## Abstract

*Objectives*: Rugby is sport with a high risk of injury. Repeated changes in exercise intensity and the high training intensity may cause to overuse injuries and long-term disability. The aim of the study was to analyze the prevalence of injuries during trainings and forms of rehabilitation procedures performed after their occurrence among elite and sub-elite rugby players. *Methods*: The data was obtained from 60 professional rugby males from France and Poland. Data were collected using paper-based recording form. It was a specially designed questionnaire which concerned specific details of the injury, including body location, type of injury, treatment and number of days off lost from playing rugby and on forms of rehabilitation procedures performed after their occurrence among elite and sub-elite rugby players. *Results*: During the study period, the overall incidence rate for injury suggested a 1.04 times more often injury occurrence (IRR = 1.04, 95% CI: 0.08; 2.00) among Polish players compared with French players; however, the distribution of injuries varied by country. The training injury incidence (TII) and incidence proportion (IP) were also higher in Poland than in France (*p* < 0.05) with the sprain as the most frequent type of injury in all rugby players. France was 7.8 times (IRR = 7.88, 95% CI: 1.29; 3.21) more likely to sustain a fracture than Poland, which much often experienced less serious injuries (bruise, rapture of muscle and ligament) (IRR = 3.02, 95% CI: 2.06; 3.98). Polish players were provided with various forms of physiotherapy while Franch players often worked with a physiotherapist with a therapeutic method (*p* < 0.005). Poland and France reported experiencing side effects after an injury and the most frequent was pain. In their opinions, the reasons influencing the effectiveness of rehabilitation are too quick return to the game and too short time of rehabilitation. *Conclusions*: The competitive level of the rugby player influences not only the frequency and type of injury occurrence, but also access to the different forms of rehabilitation. Nonetheless, the side effects occurring after injury and the causes of ineffective rehabilitation are still similar. Further studies are needed to gather significant data to accurately formulate future injury prevention protocols or recommend modifications to game laws or competition formats, aiming at players’ welfare.

## 1. Introduction

Rugby is a physically demanding sport characterized by repeated changes in exercise intensity, from low-speed activities such as standing and walking to high-intensity bouts such as sprints and tackles [1]. Also, high training volume and intensity and sport competition during matches predispose to overuse injuries [2] which may cause long-term disability, negatively affect sports participation and impact on performance and daily activities [3]. As a contact sport [4], rugby is characterized by a higher risk of injury than other team sports with similar movements [5,6] because of high loading and the unique postural stances. For example, rugby players tend to adopt an unbalanced posture posteriorly resulting in difficulty in controlling foot stability resulting in a high prevalence of lower limb specifically ankle injuries.

In countries in which rugby is popular, the toll of injury has serious public health and economic consequences [7]. Protective equipment affords the greatest potential for the prevention and mitigation of injury. Unfortunately, in comparison with players of American football and ice hockey, rugby union players are largely unprotected from impact forces [8] as rugby union prohibits hard-shell helmets and permit little or no body padding [8].

To decrease the incidence of injuries, previous studies in professional team sports [9,10,11] have recommended research follow the 4-stage van Mechelen paradigm [11] for injury prevention. This paradigm [11] requires the identification of common and serious injuries, identification of risk factors, institution of injury preventative programs, and monitoring the success of injury prevention programs. Because of rising popularity of rugby and the relatively high risk of injury associated with participation in this sport [12,13], it is imperative that epidemiological studies gather injury data to better understand and mitigate risks for the athletes [14]. Additionally, analysis of injuries occurred during training sessions is important because it affects the availability and performance of players in matches.

While incidence of injury in rugby union has been widely reported in the literature [5,15,16], attention must be given to less professional athletes. However, few authors have examined the injuries sustained by adolescent rugby players or sub-elite one [15]. The sparse data is available regarding equivalent population as analyzed in the present study [17,18,19] and to our knowledge there is no data regarding rehabilitation provided in rugby. The comprehensive knowledge about the differences in injuries obtained during trainings and rehabilitation effectiveness is required to design injury prevention models adequate to the players level. Therefore, the aim of the study was to analyze the prevalence of injuries during trainings and forms of rehabilitation procedures among elite and sub-elite rugby players. Therefore, the aim of the study was to analyze the prevalence of injuries during trainings and to investigate the differences between elite and sub-elite rugby players. Also, the aim was to describe the differences in the aspect of most common forms of rehabilitation between those two groups.

## 2. Materials and Methods

### 2.1. Participants

A total of 60 men (30 players from Poland and 30 players from France) were recruited from one Polish (P) club from Polish league and one French (F) club belonging to the French league during the season 2018–2019. Based on the popularity of discipline and its organization level in every country, Polish players were characterized as sub-elite players and French players as elite one. The participants had played professionally in rugby for an average of 5.9 ± 3.8 (P) and 5.43 ± 3.1 (F) years. The players were recruited based on the criteria: (1) regular participation in trainings during the study season; (2) being the official member of the rugby club. The exclusion criteria were health problems that prevented participation in the trainings. All players were informed of the study procedures and gave written informed consent. The study was performed in accordance with Declaration of Helsinki ethics standards and meets the ethical standards of the journal.

### 2.2. Procedures

Injury occurrence in this report was analysed only during the training sessions. Data were collected using a paper-based recording form. It was a specially designed questionnaire which concerned specific details of the injury, including body location, type of injury, treatment and number of days off lost from playing rugby. The questionnaire was based on a previous study of sports injuries [20] and the World Rugby Serious Injury Follow-up Questionnaire. It covered information on personal data, sports participation and the history of sports injuries. The athletes were asked to evaluate as precisely as possible the number of weekly training sessions and the number of hours spent per session. One part of the questionnaire concerned injury occurrence during participation in the player’s sport. This part was completed if the athlete had sustained a sports injury during his professional career. Also the anatomic location, type of injury and recovery time were registered. The second part included the questions about the rehabilitation methods and protective equipment usually used by the players. The questionnaire was pre-tested to assess its readability and understanding. The data was collected by the researcher in consultation with the trainers who had data on the presence of players in trainings.

Injury is any pain or disability that occurs during participation in rugby league match or training activities that is sustained by a player, irrespective of the need for match or training time loss or for first aid or medical attention [16]. According to the rugby union, it is any physical complaint, which was caused by a transfer of energy that exceeded the body’s ability to maintain its structural and/or functional integrity, that was sustained by a player during a rugby match or rugby training, irrespective of the need for medical attention or time-loss from rugby activities. An injury that results in a player receiving medical attention is referred to as a ‘medical-attention’ injury and an injury that results in a player being unable to take a full part in future rugby training or match play as a ‘time-loss’ injury. A brain or spinal cord injury that results in permanent (>12 months) severe functional disability is referred to as a ‘non-fatal catastrophic injury’ [21].

Training injury incidence (TII) may be expressed as the injury incidence per 1000 training hours [16]. Calculation of the training injury incidence may be undertaken by dividing the number of recorded injuries by the exposure hours, then multiplying this value by 1000 to obtain the injury incidence per 1000 training hours [22]. Exposure time was quantified as the total time the players were exposed to the possibility of injury during trainings. Over the 28-week period, rugby training volume for each player were reported by coaches based on attendance records and was recorded daily in minutes.

The epidemiologic incidence proportion (IP) is used to enable identification of the average risk per player over a specified study period [23]. The IP ideally would be reported as a percentage of risk for injury from participation in rugby league [16] and it is recommended that the time period that the IP defines is identified (i.e., season, tournament, year) [23]. Calculation of the IP may be undertaken by dividing the number of players that have sustained an injury over the study period by the number of players at risk of being injured [23].

Incidence rate (IR) per 10,000 athlete exposures (AEs) during the study period, which is defined as the number of new cases occurring during a specific period of time in a population at risk for experiencing the injury [23]. IR is calculated by dividing the total number of injuries observed in a population by a measure of exposure or person-time at risk to injury [23]. In the current study, AE was defined as 1 athlete participating in 1 training based on participation records.

### 2.3. Statistical Analysis

Statistical analyses were conducted using the STATISTICA (StatSoft, Inc. 2011, Statsoft Polska Sp. z o.o., Lodz, Poland, version 10, www.statsoft.com, accessed on 23 March 2015) to calculate averages and standard deviations. Data relating to the general characteristics of the athletes were expressed as the mean ± standard error (SE) and these data were assessed using the Student’s unpaired t-test and *p* < 0.05 was considered as statistically significant. The effect size measure of differences between the results from the beginning and after 6-month rehabilitation was verified by Cohen’s d test. It is defined as the difference between two means divided by a standard deviation for the data. Cohen classified effect sizes as small (*d* = 0.2), medium (*d* = 0.5), and large (*d* ≥ 0.8).

## 3. Results

The general characteristics of the participants are described in Table 1. The total documented athlete exposures (AEs) were 9609 h (6048 AEs of Polish players and 5376 AEs of French players). The highest percentage of players used a mouth guard as a protective equipment during trainings. But also a large group of players did not use any form of protection during trainings. (Table 1).

In total 91 injuries sustained by 60 players were reported (injury incidence rate = 7.85 injuries per 1000 players-hours; average risk rate = 1.52 per player). During the study period, the overall incidence rate for injury suggested the 1.04 times more often injury occurrence among Polish players compared with French players; however, the distribution of injuries varied by country (Table 2). The incidence rate of injury and risk rate were also higher (*p* < 0.05) in Polish players (P) than in French players (F) (Table 3). In the whole group, the head with face was the most common body region of injury, followed by spine and shoulders (Table 4 and Table 5). The statistically significant difference (*p* < 0.01) was in the frequency of the left shoulder injury which was more often noticed among P players (Table 4 and Table 5).

Regarding the type of injuries, it was found that sprain was the most frequent type of injury in all rugby players (Table 2 and Table 3). There were statistically significant differences between F and P players in the frequency of bruise, fracture and rapture of muscle and ligament injuries (*p* < 0.001; *p* < 0.02; *p* < 0.002 respectively) (Table 2 and Table 3).

In the responses regarding what was the most common form of rehabilitation, physiotherapy was cited first, followed by kinesiotherapy in both groups (Table 6). There was a significant difference (*p* < 0.001) between groups in the number of players using physiotherapy and it was higher in P players. F players more often worked with a physiotherapist with a special therapeutic method and the difference was statistically significant (*p* < 0.005) (Table 6).

The most frequent recurrent ailments after injury was pain and slightly less than 70 percent of the players reported experiencing side effects after an injury (Table 7). According to the players, the important reasons influencing the effectiveness of rehabilitation are too quick return to the game and too short time of rehabilitation (Table 7). About 26.7% of players reported using the service of the team’s physiotherapist one’s a week and it was much often noticed behaviour among F players in comparison to P players with statistically significant difference (*p* < 0.05) (Table 6).

## 4. Discussion

To our knowledge, there is little research examining simultaneously injuries occurred during training, protective strategies and rehabilitation used by the elite and sub-elite rugby players. This study illustrates some key differences in injury patterns between elite and sub-elite rugby players that may reflect competitive level, but at the same time similarity of problems noticed by elite and sub-elite players related to the return to full physical fitness after the rehabilitation process.

Rugby Union rules prohibit hard-shell helmets and allow little or no body padding [8] that is why, the highest percentage of the Polish and French players in the present study reported to use only a mouth guard as a protective equipment during training or even nothing. The lack of regulatory mandates for protective equipment in rugby could be responsible for a high general injury rate among rugby players [24] and why rugby is characterized the triple the injury rate compared to American football [8].

According to our findings, the training injury incidence and risk rate were significantly higher (*p* < 0.05) in Polish players in comparison to French players what might be due to the different status of player and thus, are subject to different rules of player care [25]. French Rugby Union implemented a specific prevention programs and law changes has notably resulted in a decrease in injuries in forwards [17]. Analyzing the most common body region of injury, the statistically significant difference between Polish and French players was noticed in the frequency of the left shoulder injury which was more often for the formers. According to research on sports injuries in professional rugby [26,27], there has been an increase in the frequency and severity of shoulder injuries among rugby players in recent years and the reason is that the game has become more aggressive and intense [26,28]. A descriptive epidemiological study performed in all players licensed in the French Rugby Union showed that shoulder injury was significantly more frequent in senior and junior players, injuries mainly occurred during a match in the middle of the season and the main mechanism was tackling [29]. On this basis, it is difficult to explain a higher frequency of shoulder injury among Polish players since the surveyed players from both Poland and France were of a similar age and had a similar training period. Probably, the professional level of the player (sub-elite/elite) should be taken as another factor determining the frequency of this injury.

Also, the statistically significant differences between French and Polish players were in the frequency of different types of injury. Polish players much often experienced injury in the form of bruise and rapture of muscle and ligament and French players usually experienced the fracture injury. Typically, soft tissue injuries account for more than 50% of all rugby-associated injuries [15,30], including musculotendinous strains and tears, in addition to ligament sprains and tears, hematomas, and contusions. Brooks and co-workers as well found that the majority of injuries (87%) involved muscles, ligaments, or joints [30].

The differences in the aspect of most common forms of rehabilitation were also evident between the two groups. For example, physiotherapy (in the other form than kinesiotherapy) was used more frequently by Polish players and working individually with a physiotherapist with a special therapeutic method e.g., McKenzie was more popular among French players. Using a service of the team’s physiotherapist even one’s a week was more common behaviour for French players than Polish players. Generally, it is considered that athletes with serious injury are more likely to seek social support with the physiotherapist what may reduce the psychological trauma experienced [31]. As it was mentioned above, French players usually experienced more often the fracture injury in comparison to Polish players which requires longer and more serious treatment and rehabilitation to return to play. Differences between the Polish and French players in the reported rehabilitation methods may also result from the fact that elite-players have access to better medical care [17,29] and rugby clubs have their own physiotherapist exclusively for players, which is not a rule in many countries.

Unfortunately, almost 70% of the players both Polish and French reported experiencing side effects after an injury and the most frequent was pain. In the opinion of the players the causes of ineffective rehabilitation were too short time of rehabilitation and too quick return to the game. The time of the athlete’s return-to-play (RTP) after an injury may have an impact on the effectiveness of the rehabilitation and the recurrent ailments. Although there is also a lack of evidence-based RTP criteria for sportspeople what results in a lack of consensus among health professionals, the different studies confirm that short recovery periods increase the reinjure risk [32].

The main strength of the current study is that it is the first of its kind analyzing simultaneously injuries, protective equipment and rehabilitation in the same sample of elite and sub-elite rugby players. Epidemiological analysis of those elements plays a pivotal role in injury prevention, providing data required for development and application of injury prevention models adequate to the players level. Next, it presents injuries sustained during trainings while other studies concentrate on the injuries mainly during matches, although it is known that particularly injuries during trainings affect the availability of players in matches.

The present study is limited in scope professional rugby players from only two countries and the sample size is relatively small and breaking down the results categories into small groups can limit the statistical power. It would be recommended to perform more longitudinal research via data collection among the players of different countries over several seasons. Moreover, the possibility of presenting the data according to playing position will be more valuable. However, during trainings players usually are on different positions, and not just on the one assigned during the match.

The intensity of training sessions increases with competition and therefore highly skilled players may experience a greater risk of injury and different one than less-skilled players. By understanding the incidence and nature of injuries in elite and sub-elite rugby players, more personalized injury prevention strategies can be implemented. Moreover, the awareness about the forms of provided rehabilitation and its efficiency among the rugby players would decrease the risk of re-injury and therefore the resultant injury incidence rates may have been lower.

## 5. Conclusions

Competitive level of the rugby player influence not only the frequency and type of injury occurrence, but also access to the different forms of rehabilitation. But yet, the side effects occurring after injury and the causes of ineffective rehabilitation are similar. Nonetheless, the side effects occurring after injury and the causes of ineffective rehabilitation are still similar. Further studies are needed to gather significant data to accurately formulate future injury prevention protocols or recommend modifications to game laws or competition formats, aiming at players’ welfare.

## Figures and Tables

**Table 1:** General characteristics of the rugby players (*n* = 60).

Variable	All the Players *N* = 60	French Players (F) *N* = 30	Polish Players (P) *N* = 30
Age (yrs.)	27.6 ± 0.6	28.03 ± 2.55	27.2 ± 3.92
Amateur career (yrs.)	14.7 ± 2.6	16.57 ± 4.31	12.87 ± 3.57
Professional career (yrs.)	5.7 ± 0.3	5.43 ± 3.15	5.9 ± 3.77
Training time (hrs per week)	1.73	1.61	1.83
Number of trainings in the season	94.2	125.2	97.2
Number of training weeks	23.5	31.3	24.5
The protective equipment	*N* (%)	*N* (%)	*N* (%)
Mouth guard	27 (45.0)	14 (46.7)	13 (43.3)
Headgear	8 (13.3)	6 (20.0)	2 (6.6)
Pad on the shoulders	4 (6.7)	3 (10.0)	1 (3.3)
No use	21 (35.0)	8 (26.7)	13 (43.3)

**Table 2:** Rugby injuries reported by rugby players (*n* = 60) by type.

The Most Common Injury Type	All the Players (*n* = 60)			French Players (F) (*n* = 30)			Polish Players (P) (*n* = 30)				
	No. of Injuries	Training Injury Incidence (TII) ^a^	Incidence Proportion (IP) ^b^	No. of Injuries	TII	IP	No. of Injuries	TII	IP	P ^c^	d
Graze	24	2.07	0.40	10	1.84	0.33	14	2.28	0.47	0.30	0.449
Bruise	22	1.90	0.37	5	0.92	0.17	17	2.76	0.57	0.001	1.877
Dislocation	16	1.38	0.27	7	1.29	0.23	9	1.46	0.30	0.57	0.173
Sprain	28	2.42	0.47	16	2.94	0.53	12	1.95	0.40	0.31	1.010
Fracture	8	0.69	0.13	7	1.29	0.23	1	0.16	0.03	0.02	1.153
Cut injury	3	0.26	0.05	2	0.37	0.07	1	0.16	0.03	0.56	0.214
Damage of internal organs	0	0.00	0.00	0	0.00	0.00	0	0.00	0.00	-	-
Rupture of muscle or ligament	14	1.21	0.23	2	0.37	0.07	12	1.95	0.40	0.002	1.612

^a^ Training injury incidence (TII) per 1000 training hours of exposure. ^b^ Incidence proportion (IP)—the average risk per player over a training period reported as a percentage of risk for injury from participation in rugby. ^c^ *p* value for the comparison of training injury incidence between French and Polish players.

**Table 3:** Incidence rates of injuries obtained among the rugby players (*n* = 60) by country.

	All the Players (*n* = 60)		French Players (F) (*n* = 30)		Polish Players (P) (*n* = 30)			
	Incidence Rate (IR) ^d^	95% CI	IR	95% CI	IR	95% CI	Incidence Rate Ratio (IRR)	95% CI
Graze	24.98	24.02, 25.94	18.60	17.64, 19.56	23.15	22.19, 24.11	1.24	0.28; 2.20
Bruise	22.90	21.94, 23.86	9.30	8.34, 10.26	28.11	27.15, 29.07	3.02	2.06; 3.98
Dislocation	16.65	15.69, 17.61	13.02	12.06, 13.98	14.88	13.92, 15.84	1.14	0.18; 2.10
Sprain	29.14	28.18, 30.10	29.76	28.80, 30.72	19.84	18.88, 20.80	1.50	6.92; 8.84
Fracture	8.33	7.37, 9.29	13.02	12.06, 13.98	1.65	0.69, 2.61	7.88	1.29; 3.21
Cut injury	3.12	2.16, 4.08	3.72	2.76, 4.68	1.65	0.69, 2.61	2.25	1.29; 3.21
Damage of internal organs	0.00	-	0.00	-	0.00	-	0.00	-
Rupture of muscle or ligament	14.57	13.61, 15.53	3.72	2.76, 4.68	19.84	18.88, 20.80	5.33	4.37; 6.29

^d^ Incidence rate (IR) per 10,000 athlete exposures (AEs) during the study period.

**Table 4:** Rugby injuries reported by rugby players (*n* = 60) by body region.

The Most Common Body Region of the Injury	All the Players (*n* = 60)			French Players (F) (*n* = 30)			Polish Players (P) (*n* = 30)				
	No. of Injuries	Training Injury Incidence (TII) ^a^	Incidence Proportion (IP) ^b^	No. of Injuries	TII	IP	No. of Injuries	TII	IP	P ^c^	d
The total number of injuries	91	7.85	1.52	42	7.72	1.40	49	7.97	1.63	0.04	0.255
Spine	14	1.21	0.23	8	1.47	0.27	6	0.98	0.20	0.55	0.500
Head and face	23	1.98	0.38	9	1.65	0.30	14	2.28	0.47	0.19	0.643
Chest	1	0.09	0.02	1	0.18	0.03	0	0.00	0.00	0.32	0.184
Abdomen	0	0.00	0.00	0	0.00	0.00	0	0.00	0.00	-	-
Right shoulder	11	0.95	0.18	4	0.73	0.13	7	1.14	0.23	0.32	0.418
Left shoulder	12	1.04	0.20	2	0.37	0.07	10	1.63	0.33	0.01	1.286
Right elbow	1	0.09	0.02	1	0.18	0.03	0	0.00	0.00	0.32	0.184
Left elbow	0	0.00	0.00	0	0.00	0.00	0	0.00	0.00	-	-
Right forearm	0	0.00	0.00	0	0.00	0.00	0	0.00	0.00	-	-
Left forearm	0	0.00	0.00	0	0.00	0.00	0	0.00	0.00	-	-
Right wrist	0	0.00	0.00	0	0.00	0.00	0	0.00	0.00	-	-
Left wrist	0	0.00	0.00	0	0.00	0.00	0	0.00	0.00	-	-
Fingers	0	0.00	0.00	0	0.00	0.00	0	0.00	0.00	-	-
Pelvis	2	0.17	0.03	2	0.37	0.07	0	0.00	0.00	0.15	0.377
Right hip	1	0.09	0.02	1	0.18	0.03	0	0.00	0.00	0.32	0.184
Left hip	0	0.00	0.00	0	0.00	0.00	0	0.00	0.00	-	-
Right tight	2	0.17	0.03	1	0.18	0.03	1	0.16	0.03	1.00	0.020
Left tight	0	0.00	0.00	0	0.00	0.00	0	0.00	0.00	-	-
Right knee	6	0.52	0.10	1	0.18	0.03	5	0.81	0.17	0.08	0.643
Left knee	4	0.35	0.07	1	0.18	0.03	3	0.49	0.10	0.31	0.316
Right crus	2	0.17	0.03	2	0.37	0.07	0	0.00	0.00	0.15	0.377
Left crus	0	0.00	0.00	0	0.00	0.00	0	0.00	0.00	-	-
Right foot	8	0.69	0.13	6	1.10	0.20	2	0.33	0.07	0.13	0.786
Left foot	4	0.35	0.07	3	0.55	0.10	1	0.16	0.03	0.31	0.398

^a^ Training injury incidence (TII) per 1000 training hours of exposure. ^b^ Incidence proportion (IP)—the average risk per player over a training period reported as a percentage of risk for injury from participation in rugby. ^c^ *p* value for the comparison of training injury incidence between French and Polish players.

**Table 5:** Incidence rates of injuries obtained among the rugby players (*n* = 60) by country.

	All the Players (*n* = 60)		French Players (F) (*n* = 30)		Polish Players (P) (*n* = 30)			
	Incidence Rate (IR) ^d^	95% CI	IR	95% CI	IR	95% CI	Incidence Rate Ratio (IRR)	95% CI
All the injuries	94.70	93.74; 95.66	78.13	77.17; 79.09	81.02	80.06; 81.98	1.04	0.08; 2.00
Spine	14.57	13.61, 15.53	14.88	13.92, 15.84	9.92	8.96, 10.88	1.50	0.54; 2.46
Head and face	23.94	22.98, 24.90	16.74	15.78, 17.70	23.15	22.19, 24.11	1.38	0.42; 2.34
Chest	1.04	0.08, 2.00	1.86	0.90, 2.82	0.00	-	0.00	-
Abdomen	0.00	-	0.00	-	0.00	-	0.00	-
Right shoulder	11.45	10.49, 12.41	7.44	6.48, 8.40	11.57	10.61, 12.53	1.56	0.60; 2.52
Left shoulder	12.49	11.53, 13.45	3.72	2.76, 4.68	16.53	15.57, 17.49	4.44	3.48; 5.40
Right elbow	1.04	0.08, 2.00	1.86	0.90, 2.82	0.00	-	0.00	-
Left elbow	0.00	-	0.00	-	0.00	-	0.00	-
Right forearm	0.00	-	0.00	-	0.00	-	0.00	-
Left forearm	0.00	-	0.00	-	0.00	-	0.00	-
Right wrist	0.00	-	0.00	-	0.00	-	0.00	-
Left wrist	0.00	-	0.00	-	0.00	-	0.00	-
Fingers	0.00	-	0.00	-	0.00	-	0.00	-
Pelvis	2.08	1.12, 3.04	3.72	2.76, 4.68	0.00	-	0.00	-
Right hip	1.04	0.08, 2.00	1.86	0.90, 2.82	0.00	-	0.00	-
Left hip	0.00	-	0.00	-	0.00	-	0.00	-
Right tight	2.08	1.12, 3.04	1.86	0.90, 2.82	1.65	0.69, 2.61	1.13	0.17; 2.09
Left tight	0.00	-	0.00	-	0.00	-	0.00	-
Right knee	6.24	5.28, 7.20	1.86	0.90, 2.82	8.27	7.31, 9.23	4.44	3.48; 5.40
Left knee	4.16	3.20, 5.12	1.86	0.90, 2.82	4.96	4.00, 5.92	2.67	1.71; 3.63
Right crus	2.08	1.12, 3.04	3.72	2.76, 4.68	0.00	-	0.00	-
Left crus	0.00	-	0.00	-	0.00	-	0.00	-
Right foot	8.33	7.37, 9.29	11.16	10.20, 12.12	3.31	2.35, 4.27	0.30	−0.66; 1.26
Left foot	4.16	3.20, 5.12	5.58	4.62, 6.54	1.65	0.69, 2.61	0.30	−0.66; 1.26

^d^ Incidence rate (IR) per 10,000 athlete exposures (AEs) during the study period.

**Table 6:** The most common rehabilitation procedures performed after injury occurrence reported by rugby players (*n* = 60).

	All the Players *n* = 60		French Players (F) *n* = 30		Polish Players (P) *n* = 30		*p* Value ^a^
	*N*	% (95%CI)	*N*	% (95%CI)	*N*	% (95%CI)	
**The most commonly recommended form of rehabilitation**							
Physiotherapy (other than kinesiotherapy)	51	85.0 (75.8, 94.2)	21	70.0 (53.3, 86.7)	30	100.0 (100.0, 100.0)	0.001
Kinesiotherapy	29	48.3 (35.4, 61.2)	11	36.7 (19.1, 54.3)	18	60.0 (42.1, 77.9)	0.07
Individual work with physiotherapist	16	26.7 (15.3, 38.1)	6	20.0 (5.4, 34.6)	10	33.3 (16.1, 50.5)	0.25
Work with physiotherapist with a special therapeutic method	10	16.7 (7.1, 26.3)	9	30.0 (13.3, 46.7)	1	3.3 (−3.2, 9.8)	0.005
**The frequency of using the service of the team’s physiotherapist**							
Once a week	16	26.7 (15.3, 38.1)	12	40.0 (27.3, 52.6)	4	13.3 (4.5, 22.1)	0.019
Every few days	10	16.7 (7.1, 26.3)	3	10.0 (2.2, 17.7)	7	23.3 (12.4, 34.2)	0.171
Few times a month	8	13.3 (4.5, 22.1)	2	6.6 (0.2, 13.0)	6	20.0 (9.7, 30.3)	0.133
Once a month	9	15.0 (5.8, 24.2)	5	16.7 (7.1, 26.3)	4	13.3 (4.5, 22.1)	0.723
Rarely	14	23.3 (12.4, 34.2)	6	20.0 (9.7, 30.3)	8	26.7 (15.3, 38.1)	0.549
Never	3	5.0 (−0.6, 10.6)	2	6.6 (0.2, 13.0)	1	3.3 (−1.3, 7.9)	0.561

^a^ *p* value for the comparison of the number of players with recommended the specific form of rehabilitation between French and Polish players.

**Table 7:** The subjective evaluation of the rehabilitation procedures reported by rugby players (*n* = 60).

	All the Players *n* = 60		French Players (F) *n* = 30		Polish Players (P) *n* = 30		*p* Value ^a^
	*N*	% (95%CI)	*N*	% (95%CI)	*N*	% (95%CI)	
**Occurrence of recurrent ailments after injury**							
Yes	41	68.3 (56.3, 80.3)	19	63.3 (50.9, 75.7)	22	73.3 (61.9, 84.7)	0.414
No	19	31.7 (19.7, 43.7)	11	36.7 (24.3, 49.1)	8	26.7 (15.3, 38.1)	0.414
**Physiological recurrent ailments after injury**							
Pain	25	41.7 (29.0, 54.4)	13	43.3 (30.5, 56.1)	12	40.0 (27.4, 52.6)	0.798
Discomfort	15	25.0 (13.8, 36.2)	7	23.3 (12.4, 34.2)	8	26.7 (15.3, 38.1)	0.770
Limitation of movement	11	18.3 (8.3, 28.3)	3	10.0 (2.3, 17.7)	8	26.7 (15.3, 38.1)	0.098
Contracture	9	15.0 (5.8, 24.2)	7	23.3 (12.4, 34.2)	2	6.6 (0.2, 13.0)	0.073
**Causes of ineffective rehabilitation**							
Too quick return to the game	17	28.3 (16.7, 39.9)	6	20.0 (9.7, 30.3)	11	36.7 (24.3, 49.1)	0.157
Too short time of rehabilitation	20	33.3 (21.1, 45.5)	10	33.3 (21.1, 45.5)	10	33.3 (21.1, 45.5)	1.00
Mismanaged rehabilitation	8	13.3 (4.5, 22.1)	6	20.0 (9.7, 30.3)	2	6.6 (0.2, 13.0)	0.133
Non-compliance the physiotherapist prescriptions	3	5.0 (−0.6, 10.6)	1	3.3 (−1.3, 7.9)	2	6.6 (0.2, 13.0)	0.561

^a^ *p* value for the comparison of the number of players with recommended the specific form of rehabilitation between French and Polish players.

## Data Availability

The data presented in this study are available on request from the corresponding author.
